# The role of E2F2 in cancer progression and its value as a therapeutic target

**DOI:** 10.3389/fimmu.2024.1397303

**Published:** 2024-05-14

**Authors:** Yang Gao, Xinjie Qiao, Zhenhui Liu, Wenzhou Zhang

**Affiliations:** ^1^ Department of Pharmacy, The Affiliated Cancer Hospital of Zhengzhou University & Henan Cancer Hospital, Zhengzhou, China; ^2^ Department of Rhinology, The First Affiliated Hospital of Zhengzhou University, Zhengzhou, China

**Keywords:** E2F2, cancer progression, therapeutic target, function, strategy

## Abstract

The E2F family of transcription factors plays a crucial role in the regulation of cell cycle progression and cell proliferation. Accumulative evidence indicates that aberrant expression or activation of E2F2 is a common phenomenon in malignances. E2F2 has emerged as a key player in the development and progression of various types of tumors. A wealth of research has substantiated that E2F2 could contribute to the enhancement of tumor cell proliferation, angiogenesis, and invasiveness. Moreover, E2F2 exerts its influence on a myriad of cellular processes by engaging with a spectrum of auxiliary factors and downstream targets, including apoptosis and DNA repair. The dysregulation of E2F2 in the context of carcinogenesis may be attributable to a multitude of mechanisms, which encompass modifications in upstream regulatory elements or epigenetic alterations. This review explores the function of E2F2 in cancer progression and both established and emerging therapeutic strategies aiming at targeting this oncogenic pathway, while also providing a strong basis for further research on the biological function and clinical applications of E2F2.

## 1 Introduction

The E2F family is an important transcription factor (TF) which plays a critical regulatory role in the cell cycle. E2F family members are defined by their DNA binding domain (DBD), and to date, eight members, contain E2F1- E2F8, have been identified, which encode for nine different gene products (1, 2). E2F transcription factor 2 (E2F2) is a transcription factor belonging to the E2F family. E2F2 is involved in biological processes such as cell cycle regulation, cell differentiation and apoptosis. When the cell enters the S phase, E2F2 can combine with DP1 to form a complex that promotes DNA synthesis and the normal progression of the cell cycle (3). In addition, E2F2 is also involved in regulating the transcriptional activities of many other genes, such as BAX, p73, p16INK4a, etc. (4). E2F2 plays an important role in a variety of physiological and pathological conditions, including biochemistry, molecular biology, cell and developmental biology, and oncology and so an. In cancer, overexpression of E2F2 has been found in various types of cancer, and the abnormal expression of E2F2 is often associated with the occurrence, metastasis and recurrence of tumors.

Overall, E2F2 is a multifaceted regulatory factor that participates in numerous physiological and pathological processes and involves various types of signaling pathways. In recent years, extensive research has confirmed the significant role of E2F2 in tumor progression, which can exert pro-cancerous or anti-cancerous effects depending on its specific context. Additionally, E2F2 has been shown to be associated with the efficacy of some anti-tumor drugs, indicating that it may serve as a new candidate for targeted cancer therapy. Therefore, this review summarizes the functions and roles of E2F2 in cancer progression, further exploring its contributions to tumor development and identifying potential therapeutic targets.

## 2 Discovery, structure, and distribution of E2F2

E2F2 is a member of the E2F family of transcription factors, which play important roles in the regulation of cell cycle, DNA replication, and differentiation. The discovery of E2F2 dates back to 1992 when it was first identified as a protein that binds to the adenovirus E2 promoter. E2F2 belongs to the E2F family of transcription factors, which contain a highly conserved DNA-binding domain known as the E2F DNA-binding domain. This domain consists of a pair of zinc fingers that specifically recognize and bind to DNA sequences in target gene promoters. E2F2 also contains a transactivation domain, which allows it to activate or repress gene expression. E2F2 is ubiquitously expressed in mammalian tissues, with high levels detected in proliferating cells such as embryonic tissues and cancer cells. In humans, the E2F2 gene is located on chromosome 1p36.13, and mutations in this gene have been associated with various types of cancer. E2F2 has been shown to interact with a variety of proteins involved in cell cycle regulation and DNA repair, including Rb, p53, and BRCA1 (5–8). E2F2 forms complexes with DP1 and DP2, members of the DP family of transcription factors, to regulate gene expression (9). The E2F2/DP1 or E2F2/DP2 complex can function either as a transcriptional activator or a transcriptional repressor, depending on the specific context and interacting partner proteins (10, 11). For example, the E2F2/DP1 complex activates the expression of genes required for the G1-to-S phase transition, while the E2F2/HDAC1 complex represses the expression of genes involved in cell cycle progression (12–14).

In summary, E2F2 is a ubiquitously expressed transcription factor that plays important roles in the regulation of cell cycle progression, DNA replication, and differentiation. Its structure and distribution have been well studied, and it forms complex interactions with multiple partner proteins to orchestrate gene expression programs in the cell.

## 3 Functions of E2F2 in specific cancer types

In the process of cancer progression, malignant proliferation of tumor cells is the main factor for its occurrence and development, and the disorder of cell cycle process is the root cause of malignant proliferation of cancer. Transcription factor E2F2 plays an important role in regulating cell cycle progression. Besides the limited evidence demonstrating that E2F2 could suppress tumor progression, an increasing number of experiments indicated that E2F2 was closely related to the occurrence of multiple cancer types. Based on the key role of E2F2 in the occurrence of cancer through the regulation of cell cycle process, further research on the regulatory mechanism of E2F2 in the process of tumor formation may provide new research ideas in the control of tumor growth and the development of novel anti-tumor drugs. In recent decades, in-depth research on E2F2 in cancer has elucidated E2F2 functions in tumor-cell metabolism, anti-angiogenesis, metastasis, therapy resistance, and tumor immunoediting (**Table 1**).

**Table 1:** The effect of E2F2 on cancer.

Effect on cancer	Type of cancer	Function or consequence
Promotes cancer	Breast cancer	Increase latency, impact the progression and metastasis, inhibit cell-matrix adhesion
	Hepatocellular carcinoma	Promote proliferation and metastasis, inhibit apoptosis, poor prognosis
	Prostate cancer	Promote proliferation, colony formation, migration and invasion
	Lung cancer	Promote proliferation and invasion
	Glioblastoma	Promote the growth and metabolism
	Ovarian cancer	Worse overall survival
	Colorectal cancer	Reciprocal feed-forward with B-Myb
	Gastric cancer	Promote migration and invasion, autophagy, preservation of the immunodominant status
	Pancreatic ductal adenocarcinoma (PDCA)	Promote angiogenesis and cell growth
	Neuroblastoma	Reduce overall survival
	Osteosarcoma	Promote proliferation
Inhibits cancer	Glioblastoma	Enhance the anti-cancer effect of p53
	Colorectal cancer	Inhibit immune cell infiltration, improve prognosis
	T cell lymphomagenesis	Inhibit tumor progression
	skin and oral cancer	Limiting proliferation in response to Myc

### 3.1 Breast cancer

For women, breast cancer has the first place in cancer incidence worldwide and is the twice place in mortality worldwide by 2023 (15, 16). Several studies have shown that E2F2 is overexpressed in breast tumors compared to normal tissue (17, 18).

The mortality may always find links to the metastasis to distant sites in human breast cancer. Aliccia Bollig-Fischer found that E2F2 aberrant expression impacts the progression and metastasis of breast cancer cells, E2F2 expression regulated by human epidermal growth factor receptor 2 (HER2) oncogene could impact the cell-matrix adhesion function, with potential consequences for metastatic colonization (19). Transcriptional regulation of E2F2 plays a crucial role in HER2 overexpression, the overexpression of HER2 in breast tumors is associated with bad prognosis (20). Yuwanita reported that E2F2 loss results in increased metastasis in Myc-driven breast cancer through a PTPRD dependent mechanism potentially (21). E2F2 knockout mice have reduced expression of genes associated with the epithelial-mesenchymal transition (EMT) (22). In addition, E2F2 is related to breast cancer stem cells (BCSCs). Inhibition of E2F2 could reduce the proportion of CD24^-^/CD44^+^ cells and the expression levels of SOX2 and OCT4, thus inhibiting the self-renewal, proliferation and invasion ability of BCSCs and inhibiting the growth of breast cancer tumors (23). Moreover, E2F2 was also correlated with a patient’s response to PARP inhibition therapy (24). Therefore, E2F2 may be a potential target for breast cancer therapy.

### 3.2 Hepatocellular carcinoma

Liver cancer remains a global health challenge, with an estimated incidence of >1 million cases by 2025. Hepatocellular carcinoma (HCC) is the most common form of liver cancer and accounts for ~90% of cases (25). It is the sixth most common cancer in incidence and the third leading cause of cancer death among all malignancies (16). Studies have shown that the expression level of E2F2 was generally upregulated in HCC. High expression of E2F2 was associated with poor prognosis in patients (26). Similarly, Gene Expression Omnibus (GEO) data and TCGA analysis shown that E2F2 mRNA was highly expression in HCC tissue and was significantly correlated with tumor status, histological grade, and clinical stage (27). Elevated E2F2 could be a promising independent prognostic biomarker and therapeutic target for HCC (28). Therefore, E2F2 is inextricably related to the progression of HCC.

Several studies already proved that high expression of E2F2 serves as a driving force for the development of HCC (29). Down-regulating the expression of E2F2 could inhibit the proliferation of HCC cells (30). E2F2 overexpression significantly inhibited HCC cell apoptosis in a p53-dependent manner (31). Fang et al. found that HCC cell metastasis could be restrained by down-regulating of the expression of E2F2 and ECT2 (32). Activating OCT1-E2F2 signaling would intensify sorafenib and lenvatinib resistance in HCC (33). Moreover, E2F2 overexpression plays a central role in dysregulation of the cell cycle in HCC. Overexpressed E2F2 is a crucial center of cell cycle regulation and is significantly associated with patients’ poor prognosis (34).

Long non-coding RNAs (lncRNAs) have been confirmed to influence the development of HCC. Minzhen et al. found that lncRNA PRR34-AS1 could promote HCC development via modulating Wnt/β-catenin pathway by upregulating E2F2 and SOX12 (35). In addition, lipid metabolism rearrangements in nonalcoholic fatty liver disease (NAFLD) contribute to disease progression. NAFLD has emerged as a major risk for HCC. Francisco et al. found that activation of the E2F1-E2F2-CPT2 axis provides a lipid-rich environment required for hepatocarcinogenesis, E2F2 deletion confers protection against hepatocarcinogenesis by preventing lipid storage (36). Altogether, these results indicate that E2F2 might be vital to HCC development.

### 3.3 Prostate cancer

Prostate cancer ranks first in the number of the estimated new cases and as the second leading cause of cancer-related death in men in the America (16). The incidence and fatality rate of prostate cancer is rising continuously worldwide (37). Explore the underlying reasons and new preclinical prevention or treatment measures is urgently needed. Li et al. demonstrated that E2F2 regulated DLEU2 expression by directly binding to DLEU2 promoter. The overexpression of E2F2 in prostate cancer cells contributed to upregulation of DLEU2, then facilitated prostate cancer progression, including proliferation, colony formation, migration, and invasion (38).

E2F2 has been well-characterized as a regulator of the G1-to-S-phase transition, it has been confirmed that it may be novel therapeutic candidate for prostate cancer given its ability to induce cell-cycle arrest and inhibit cell growth. The tumor suppressor PTEN is the most frequently inactivated gene in prostate cancer. PTEN-mediated G1 cell-cycle arrest in LNCaP prostate cancer cells is associated with altered expression of E2F2 (39). Dong et al. found that let-7a acts as a tumor suppressor in prostate cancer by down-regulating E2F2 and CCND2, further study shown that the 3’UTR of E2F2 and CCND2 could directly bind to let-7a and then induce cell cycle arrest at the G1/S phase (40).

Additionally, E2F2 activity controls the transcriptional regulation of a myriad of genes encoding key proteins involved in nucleotide biosynthesis, DNA replication and DNA repair (41, 42). Mohaddase Hamidi et al. shown that targeting E2F1/E2F2 elicited DNA damage during S phase, leading to premature CDK1 activation and compromised prostate cancer cells viability (43).

### 3.4 Lung cancer

Lung cancer is one of the most aggressive tumors with very low life expectancy (44, 45). E2F2 acts as an activator is closely related to clinical stage and tumor size (46). Analysis of ONCOMINE, GEPIA, Kaplan-Meier Plotter and cBioPortal databases of E2F2 in patients with Lung cancer shown that the expression level of E2F2 was higher in lung adenocarcinoma and squamous cell lung carcinoma tissues than in lung tissues (47). The expression level of E2F2 was correlated with advanced tumor stage. Survival analysis using the Kaplan-Meier Plotter database revealed that the high transcription level of E2F2 were associated with low relapse-free survival (RFS) in all of the patients (47).

In non-small cell lung cancer (NSCLC), multiple studies have shown that up-regulation of E2F2 could promote the progression of lung cancer (48). The activating E2F2 Signaling would promote proliferation and invasion (49, 50). Mechanically, E2F2 may play a key role in the pathological mechanism of NSCLC progression through Circ_0109320/miR-595/E2F2 axis (51). In addition, Zhang et al. reported that inhibition of E2F2 transcription activity could reduce the activity of PI3K/AKT signaling pathway, and thus potentially inhibit the occurrence of lung cancer (52).

Lung adenocarcinoma causes severe cancer death worldwide. Du et al. constructed an E2F2-centered co-expression network and identified several key modules and networks overrepresented in Lung adenocarcinoma. Function analysis revealed that E2F2 overexpression accelerated cell growth, cell cycle progression and cell motility whereas E2F2 knockdown inhibited these malignant phenotypes. Mechanistic investigations uncovered that E2F2/B-Myb/FOXM1 from the Lung adenocarcinoma transcription regulatory network exhibited positive expression correlation, associated with each other, mutually transactivated each other, and regulated similar downstream gene cascades, hence constituting a consolidated core transcription regulatory circuitry (53).

### 3.5 Glioblastoma

Glioblastoma is a type of brain cancer that originates from the glial cells in the brain, which is the most aggressive and common form of primary brain tumor in adults. Research has shown that E2F2 may play a role in glioblastoma. Abnormal expression and activity of E2F2 have been observed in glioblastoma cells, suggesting that it may contribute to the development and progression of the disease. RNAi-mediated knockdown of E2F2 could inhibit tumorigenicity of human glioblastoma cells (54). However, for another, Mitlianga et al. found that co-expression of E2F2 and p53 enhances the anti-cancer effect of p53 in Glioblastoma cells (55). Unquestionable integrity, E2F2 is involved in gliomagenesis and could be explored as a potential therapeutic target in malignant gliomas.

Numerous microRNAs are strongly implicated in the malignancy of glioblastoma. Song et al. found that Let-7b, a member of the Let-7 microRNA family, could inhibit the malignant behavior of glioma cells and glioma stem-like cells via downregulation of E2F2 (56). Furthermore, by directly targeting E2F2, miR-125b could regulate the proliferation and miR-218 could inhibit the growth and metabolism of human glioblastoma cells (57, 58). In addition, peroxisome proliferator-activated receptor alpha (PPARalpha) is a member of the nuclear hormone receptor superfamily and functions as a transcription factor. Research has shown that PPARalpha could induce miR-214 expression by downregulation of its target E2F2. Subsequently, the overexpression of miR-214 inhibits Glioblastoma cells growth *in vitro* and *in vivo* by inducing cell cycle arrest in G0/G1 (59). Collectively, these data uncover a crucial role for microRNAs-E2F2 pathway in controlling the development of glioblastoma.

### 3.6 Ovarian cancer

Ovarian cancer (OC) is the fifth most common gynecological malignancy and one of the most lethal cancers in females (16). The lack of effective biomarkers is believed to be an important reason for the high lethality rate of ovarian cancer.

Previous studies indicated that E2F2 was a significantly upregulated transcription factor in ovarian cancer epithelial cells and among the OC samples, and simultaneously, high E2F2 expression was negatively associated with tumor stage and outcome among OC patients (60). E2F2 might serve as potential prognostic biomarkers and targets for OS based on the comprehensive analysis between the E2F2 and the pathogenesis and progression of OC (1).

LBX2‐AS1 was a novel lncRNA that promoted the progression of OC by inhibiting miR-455–5p and miR-491–5p. Jian Cao et al. showed that inhibition of these two miRNAs or the forced expression of E2F2 counteracted the effect of LBX2‐AS1 knockdown on the malignancy of OC cells (61). In addition, circRNAs have gained significant attention in cancer research. research has shown that circE2F2 promoted OC cell proliferation, metastasis, and glucose metabolism by stabilizing the E2F2 mRNA via binding to the HuR protein (62). These findings suggest an essential regulatory role of E2F2 on ovarian carcinogenesis.

### 3.7 Colorectal cancer

Emerging evidence has revealed that E2F2 participates in the tumorigenesis and progression of various tumors, although its function in colorectal cancer (CRC) is not yet fully elucidated (63, 64). On the one hand, studies have indicated that the expression of E2F2 was significantly elevated in colon cancer tissues compared to normal colon tissues, implicating E2F2 as a potential diagnostic biomarker for CRC (2). B-Myb could accelerate CRC progression via reciprocal feed-forward transactivation of E2F2 (65). On the other hand, study illustrated that E2F2 was significantly downregulated in CRC samples. Reduced E2F2 expression in CRC was prominently correlated with N, M stage and pathological stage. Decreased E2F2 expression was unfavorable for overall survival (OS), disease free survival (DFS), disease specific survival (DSS) and progress free interval (PFI) (64). Decreased E2F2 expression is closely associated with poor prognosis and immune cell infiltration in CRC, suggesting its potential as an independent prognostic biomarker and a therapeutic target for CRC (64). Furthermore, Li et al. have found that miR-31 regulated the proliferation of CRC cells by targeting E2F2, and E2F2 acted as a tumor suppressor in CRC by repressing the expression of survivin and regulating the expression of CCNA2, C-MYC, MCM4 and CDK2 (66).

### 3.8 Gastric cancer

Gastric cancer (GC) is a malignant tumor that originates from the epithelial cells of the stomach. It is one of the most common malignancies and a leading cause of cancer-related deaths worldwide. It can be caused by multiple factors, including genetic mutations, environmental factors, and lifestyle choices.

Bioinformatic analysis using multiple databases revealed that the level of E2F2 was highly expressed in both GC tissues and cells compared with that in normal gastric tissues and cells. High E2F2 expression was associated with the characteristics of invasive tumors and poor prognosis. Study had shown that E2F2 play a key role in the regulation of PI3K/Akt/mTOR-mediated autophagy and the downstream processes of cell migration and invasion (67). E2F2 also had potential modulatory effects on tumor immunity. CIBERSORTx analysis of the proportion of tumor-infiltrating immune cells (TICs) revealed that immune cells are correlated with E2F2 expression, suggesting that E2F2 might be responsible for the preservation of the immunodominant status for gastric cancer (68).

miRNA is a class of short chain non-coding RNA molecules that can participate in intracellular gene regulation after transcription and play an important role in the process of tumor occurrence and development (69). Researches have shown that E2F2 expression is up-regulated in GC tissues and is inversely proportional to the level of miR-31, downregulated miR-31 level associates with poor prognosis of gastric cancer and its restoration suppresses tumor cell malignant phenotypes by inhibiting E2F2 (70). Wen et al. confirmed that miR-26a could improve the sensitivity of GC cells to cisplatin-based chemotherapies through targeting NRAS and E2F2 (71). H19 could be used as a biological indicator for diagnosing GC and predicting patients’ poor prognosis. A study revealed that H19 could promote GC to proliferate and invade through miR-138/E2F2 axis (72). Therefore, miRNA and E2F2 have broad application prospects and development space in the research field of gastric cancer. In the future, with the in-depth understanding of the functional mechanism and regulatory network between miRNA and E2F2, their importance in tumor therapy and diagnosis will be further confirmed and played.

### 3.9 Other

E2F2 plays a significant role in a variety of other tumors as well. Pancreatic ductal adenocarcinoma (PDAC) is one of the most aggressive tumors all over the world. M2 macrophage-derived exosomes promoted angiogenesis and growth of PDCA cells by targeting E2F2 (73). In mice xenografts of neuroblastoma cells stably expressing doxycycline-inducible DOT1L shRNA, ablating DOT1L expression with doxycycline significantly reduced ODC1 and E2F2 expression, reduced tumor progression, and improved overall survival (74). miR-218 suppresses the proliferation of osteosarcoma through downregulation of E2F2 (75). Gliomas are highly resistant to conventional treatment. Co-expression of E2F-2 and p53 enhances the anti-cancer effect of p53 in gliomas (55).

E2F2, in addition to its capacity to facilitate tumor progression, has also been implicated in the suppression of tumor growth through specific mechanisms. Study has demonstrated that E2F2 inactivation could cooperate with transgenic expression of Myc to enhance tumor development in the skin and oral cavity, perhaps by limiting proliferation in response to Myc (76). Rene et al. used a bitransgenic mouse model of Myc-induced T cell lymphomagenesis and analyzed tumor progression in mice deficient for E2F1, E2F2, or E2F3. Surprisingly, the targeted inactivation of E2F1 or E2F3 has no significant effect on tumor progression while the loss of E2F2 accelerates the development of lymphoma (77).

## 4 Strategies to inhibit E2F2 function

A multitude of studies have repeatedly shown that blocking E2F2 activity limits tumor cell proliferation and viability due to the promoting role of E2F2 in tumor progression. Here, we summarize several strategies that have been used to either alter or utilize E2F2 activity in preclinical settings to target cancer cells (**Table 2**).

**Table 2:** The strategies to inhibit E2F2 function.

Strategies	Mechanism or consequence
Small molecules	disrupt the binding, promote the degradation or interfere the protein-protein interaction.
Molecular glues	catalyze degradation
Peptide inhibitors	interfere E2F2 function
RNA interference	target and degrade E2F2 mRNA, reduce E2F2 protein levels and inhibit function
Antisense oligonucleotides	inhibit the translation of E2F2 mRNA into protein
Gene editing techniques	target and disrupt the E2F2 gene

Small molecules could be designed through high-throughput screening to specifically inhibit E2F2 function. These inhibitors could disrupt the binding of E2F2 with its target genes, promote the degradation or interfere the protein-protein interaction. Molecular glues are a class of small-molecules which could catalyze the rapid degradation of previously inaccessible proteins through a proximity-induced interaction between the target protein and E3 ligases. Study have indicated that bufalin as molecular glue markedly promoted E2F2-ZFP91 complex formation, thereby leading to E2F2 polyubiquitination via K48-linked ubiquitin chains for degradation (78). Peptide inhibitors that specifically inhibit E2F2 activity were designed using synthetic or natural peptide molecules. These peptides are able to interact with E2F2 and interfere its function. Several peptides isolated from random peptide phage display libraries have been designed to interfere with the ability of E2Fs including E2F2 to bind DNA. Incubation of human tumor cells with these peptides led to an inhibition of cell proliferation and induction of apoptosis (79). RNA interference (RNAi) could be used to specifically target and degrade E2F2 mRNA, leading to reduce E2F2 protein levels and inhibited function. Trang Nguyen-Vu et al. found that knockdown of E2F2 expression resulted in a significant disruption of estrogen receptor positive breast cancer cell proliferation (80).

In addition, there are several strategies that could be attempted to inhibit the function of E2F2. Antisense oligonucleotides (ASOs) were short synthetic strands of nucleic acids that could bind to specific RNA molecules, including E2F2 mRNA. By binding the E2F2 mRNA, ASOs could block its translation into protein, effectively inhibiting E2F2 function. Gene editing techniques such as CRISPR-Cas9 could be used to specifically target and disrupt the E2F2 gene. E2F2 activity is regulated by various upstream signaling pathways and proteins. Targeting these regulators, such as cyclin-dependent kinases (CDKs) or retinoblastoma protein (RB), can indirectly inhibit E2F2 function. Further research and validation are required to assess the efficacy and safety of these strategies before they can be utilized for therapeutic purposes.

## 5 Conclusion and perspectives

As a gene family associated with the regulation of many cellular functions such as cell cycle, apoptosis, differentiation, and stress response, the E2F transcription factor family plays a critical role in cell regulation. Many signals can regulate the activity of E2F family members, and E2F family members can in turn regulate multiple different target genes, thus affecting different cell functions and processes. Therefore, tight control of the expression of E2F family members plays an important role in the physiological balance of cellular tissue, and once this is disrupted, it may lead to functional disorders or even tumor occurrence. In such a regulatory network, E2F2 occupies a central position and plays an irreplaceable role in regulating intracellular signals.

Like other members of the E2F family, E2F2 usually binds to the promoter region of genes to control their expression, playing a crucial role in the regulation of the cell cycle. In recent years, with the deepening research on the relationship between the E2F family and cancer, the function of the E2F2 is no longer limited to the cell cycle. In the human microenvironment, E2F2 exhibits a multifaceted and nuanced role, interacting with a myriad of molecular pathways and cellular processes. Its intricate involvement in the regulation of cell proliferation, apoptosis, and DNA repair mechanisms underscores its significance in maintaining genomic stability and cellular homeostasis. Furthermore, E2F2’s complex interaction with other transcription factors and its responsiveness to various signaling contribute to its diverse functions in development, tissue regeneration, and disease pathogenesis, including cancer.

However, similar to the majority of genes, E2F2 exhibits a dual functionality within living organisms. It is capable of not only promoting processes such as cell proliferation, apoptosis, and inflammation but also inhibiting these processes. E2F2 facilitates the transition of cells into the S phase, promoting DNA replication and cell proliferation. However, excessive E2F2 activation could disrupt the cell cycle and inhibit cell growth. E2F2 could both induce apoptosis, which is crucial for tissue homeostasis and cancer prevention, and suppress apoptosis, potentially leading to the survival of abnormal cells and tumor growth. Additionally, E2F2 can modulate the inflammatory response, which is a defense mechanism against infection and injury, but its overactivation can result in tissue damage and chronic diseases. E2F2 can also inhibit inflammation to prevent excessive injury.

E2F2 has been shown to play a dual role in cancer progression. On the one hand, it can act as a tumor suppressor by inhibiting the proliferation of cancer cells and promoting apoptosis. On the other hand, E2F2 can also promote tumor growth and metastasis by regulating the expression of genes involved in cell cycle progression, angiogenesis, and epithelial-to-mesenchymal transition (EMT). The context-dependent function of E2F2 in cancer makes it a challenging but promising target for therapeutic intervention.

The biological function of E2F2 in cancer is complex, with its dysregulation affecting various pathways and processes. It has been shown to influence metastasis, cancer stem cell properties, apoptosis, drug resistance, cell cycle progression, and transcriptional regulation of key cancer-related genes. In different cancer types, E2F2 interacts with factors such as PTPRD, p53, OCT1, let-7a, and B-Myb, and is involved in pathways including Wnt/β-catenin and PI3K/AKT. Its role as a tumor suppressor or oncogene appears to be context-dependent, highlighting the need for a nuanced understanding of its functions in cancer biology. However, we believe that the current findings are just the tip of the iceberg of its functions. There is still a need for further research on its extensive functions and how it regulates different genes, mutually promotes and inhibits various genes, and complex regulatory networks.

Although the specific mechanisms and roles of E2F2 in tumorigenesis have not been fully elucidated yet, the existing research results have already demonstrated the importance of E2F2 as a therapeutic target. Studies have shown that inhibiting E2F2 expression and reducing the expression level of E2F2 protein can suppress tumor occurrence, growth, metastasis, and recurrence. Therefore, screening for drugs that target E2F2 provides new evidence for the development of anti-cancer drugs. However, there are still many difficulties regarding drug development targeting E2F2. Such as how to use PROTAC technology to develop small molecule drugs that are more selective, less side effect, and stronger potency? What is the binding site of E2F2 small molecule inhibitors with E2F2? How to modify the developed drugs to reduce their side effects, improve their selectivity and efficacy? What is the antigenic epitope recognized by anti-E2F2 antibodies? Are antibody drugs better than E2F2 small molecule inhibitors? Will knocking out or inhibiting E2F2 have any adverse effects on the body? Answering these questions will help to find candidate drugs that regulate the function of E2F2 better.

With a better understanding of E2F2’s contributions to cancer development, researchers may be able to develop novel diagnostic tools, therapeutic targets, and treatment strategies. By exploring the complex interplay between E2F2 and other cellular factors, it may be possible to identify new pathways for intervention and improve patient outcomes. Ultimately, the ongoing investigation of E2F2’s role in cancer may pave the way for more effective and personalized treatment approaches, offering hope for cancer patients in the future.

## Author contributions

YG: Writing – original draft, Writing – review & editing. XQ: Writing – review & editing, Formal analysis. ZL: Writing – original draft. WZ: Writing – review & editing.
